# Are prophylactic haematopoietic growth factors of value in the management of patients with aggressive non-Hodgkin's lymphoma?

**DOI:** 10.1038/sj.bjc.6601708

**Published:** 2004-03-02

**Authors:** A Hackshaw, J Sweetenham, A Knight

**Affiliations:** 1 Cancer Research UK & UCL Cancer Trials Centre, Stephenson House, London NW1 2ND, UK; 2 University of Colorado Health Sciences Center, Denver, USA; 3 Evicom Ltd, Twickenham TW1 2AA, UK

**Keywords:** non-Hodgkin's lymphoma, granulocyte colony-stimulating factor, cost-effectiveness, meta-analysis

## Abstract

Combination chemotherapy used to treat patients with aggressive non-Hodgkin's lymphoma is associated with neutropenia and subsequent infection, hospital admission and treatment delays. Haematopoietic growth factors (HGF) can prevent neutropenia and improve quality of life. We undertook a meta-analysis of six randomised and one nonrandomised trials to quantify the effect in previously untreated patients, and a simple cost-effectiveness analysis. The trials compared HGF plus chemotherapy with chemotherapy alone. In total, there were 779 patients aged between 15 and 82 years. Haematopoietic growth factors was associated with a statistically significant 44% reduction in the incidence of severe neutropenia (neutrophil count <0.5 × 10^9^ l^−1^), a 60% reduction in the number of hospital admissions due to infection, an 80% reduction in the number of patients who had a treatment delay due to neutropenia and a 50% reduction in hospital stay. These data together with UK G-CSF drug costs were combined to develop a simple cost-effectiveness model, based on direct costs. Given the current cost of G-CSF, it would only be cost-effective among patients in which high rates of hospital stay due to neutropenia or infection are expected. Alternatively, if the cost could be reduced then all patients may be able to obtain the benefits. However, the evidence that prophylactic HGFs are clinically worthwhile is clear.

## BACKGROUND

Intensive chemotherapy can be highly effective in the treatment of aggressive non-Hodgkin's lymphoma (NHL). However, this form of treatment is associated with neutropenia that can result in infection and subsequent hospital admission, treatment delays and chemotherapy dose reduction. Haematopoietic growth factors (HGF) can be used to prevent neutropenia and its consequences in untreated patients with advanced NHL. We here present a meta-analysis based on the controlled trials of the clinical effectiveness of such growth factors, when used as a primary prophylaxis. We also present a simple cost-effectiveness analysis.

## PATIENTS AND METHODS

### Clinical effectiveness

A systematic review of the literature was performed to identify clinical trials that compared HGF plus chemotherapy with chemotherapy alone. The literature search covered several medical databases; Medline, Embase, Cancerlit, Cochrane library, the UICCR Trials Register and the publication databases of the European Haematology Association and the American Society of Hematology. Keywords used were ‘lymphoma’, ‘growth factors’, ‘G-CSF or GM-CSF’ and ‘trial’.

The analyses presented here were based on the six randomised controlled trials (Pettengell *et al*, 1992; Gerhartz *et al*, 1993; Aviles *et al*, 1994; Fridik *et al*, 1997; Gisselbrecht *et al*, 1997; Zinzani *et al*, 1997) and one nonrandomised trial (Bertini *et al*, 1996), which assessed the use of HGF in patients with aggressive NHL who had not been treated previously. All but one trial used granulocyte colony-stimulating factor (G-CSF), the other trial used granulocyte-macrophage-CSF (GM-CSF) (Gerhartz *et al*, 1993). The dose was specified as 5 *μ*g kg day^−1^ in five trials (Aviles *et al*, 1994; Bertini *et al*, 1996; Fridik *et al*, 1997; Gisselbrecht *et al*, 1997; Zinzani *et al*, 1997), 5.6 *μ*g kg day^−1^ in one trial (Gerhartz *et al*, 1993) and 230 *μ*g m^−2^ in another trial (Pettengell *et al*, 1992).

Information on the following outcomes were obtained for each treatment group from each published report, where available:

- the incidence of severe neutropenia (neutrophil count <0.5 × 10^9^ l^−1^)
- the incidence of severe or clinically important infections. The definition of this varied between the trials and are given in the Footnote to Table 2.
- the proportion of patients admitted to hospital
- the average length of stay in hospital
- the proportion of patients who had their chemotherapy treatment delayed
- the proportion of patients with complete (or complete/partial) tumour remission
- the proportion of patients surviving to 2, 2.5 or 5 years

For each outcome and trial, the relative risk and 95% confidence interval (CI) was calculated by comparing the proportion (or incidence) in the G-CSF group with the proportion (or incidence) in the control (no G-CSF) group. A statistical test for heterogeneity was performed for each outcome to assess whether the relative risks were significantly different between the trials (Whitehead and Whitehead, 1991). In the absence of statistically significant heterogeneity, indicating consistency between the trial results, the pooled relative risk was obtained by taking an average of the log relative risks each weighted by its standard error (Whitehead and Whitehead, 1991).

### Cost-effectiveness

A measure of financial cost was taken as the cost of hospitalisation per patient associated with a febrile neutropenic event. A sensitivity analysis was based on varying (i) the percentage of patients who, if not given HGF, would be hospitalised (this is the same as the chance of a single patient being hospitalised) and (ii) the number of times each patient could be hospitalised during five treatment cycles (the number of cycles can vary in practice, usually from 4 to 6, so we reported results for five cycles). We estimated the percentage reduction in the published list price of G-CSF that would be needed in order for the health service cost to be cheaper than if it were not used. The analysis was also performed assuming that the dose of G-CSF could be reduced from the standard dose of 5 to 2 *μ*g kg day^−1^ (a clinical trial has suggested that the lower dose has a similar effect on neutropenia as the standard dose (Toner *et al*, 1998)).

Two results from the meta-analysis of the clinical outcomes were used:

- The reduction in hospital admission due to infection
- The reduction in length of stay

The cost parameters were as follows:

- The cost of G-CSF per patient was taken as £4406 (using a typical list price of £3750, assuming £75 (British National Formulation, 2002) per day over 10 days in each of the five treatment cycles and increased by 17.5%, Value Added Tax).
- The cost of hospitalisation for a patient with a neutropenic event was taken as £2750; estimated using the figure of £2290 (1996 costs, Office of Health Economics, 1998) and increased by 3% per year to possibly reflect current costs (in 2002) after inflation.
- The cost of chemotherapy was not included since this will be the same regardless of whether the patient received G-CSF or not.

The cost per patient not given G-CSF is estimated as the percentage of patients hospitalised × cost of hospitalisation × number of cycles each patient is admitted for. The cost per patient given G-CSF is estimated by the same formula but the percentage of patients hospitalised is reduced by the relative risk associated with hospitalisation and the reduction in the length of stay (obtained from the meta-analysis of the clinical trials) and the cost of G-CSF is added.

## RESULTS

### Clinical effectiveness

Table 1

**Table 1:** General information on the controlled trials of G-CSF in patients with high-grade non-Hodgkin's lymphoma

**Trial reference^a^**	**Country**	**Age range of patients, years (mean age)**	**Chemotherapy**	**Number of patients given G-CSF^b^**	**Number of patients not given G-CSF**
Pettengell *et al* (1992)	UK	16–71 (52)	VAPEC-B^c^	41	39
Gerhartz *et al* (1993)^b^	Germany	15–73 (50)	COP-BLAM^d^	87	85
Aviles *et al* (1994)	Mexico	18–65 (51)	ESAP, m-BECOD, MVPP-Bleo^e^	20	22
Bertini *et al* (1996)	Italy	65–80 (71)	P-VEBEC^f^	46	54
Fridik *et al* (1997)	Austria	18–75 (52)	CEOP/IMVP-Dexa^g^	38	36
Gisselbrecht *et al* (1997)	France	16–55 (38)	LNH-84^h^	82	80
Zinzani *et al* (1997)	Italy	60–82 (69)	VNCOP-B^i^	77	72

a All were randomised except Bertini *et al* (1996).

b The trial by Gerhartz *et al* (1993) used GM-CSF.

c VAPEC-B (vincristine, adriamycin, prednisolone, etoposide, cyclophosphamide, bleomycin).

d COP-BLAM (cyclophosphamide, vincristine, prednisolone, bleomycin, doxorubicin, procarbazine).

e ESAP (etoposide, Solu-Medrol, cytosine arabinoside, cis-platinum); m-BECOD (methotrexate, bleomycin, epirubicin, cyclophosphamide, vincristine, dexamethasone).

f P-VEBEC (epirubicin, cyclophosphamide, etoposide, vinblastine, bleomycin, prednisone).

g CEOP/IMVP-Dexa (cyclophosphamide, epirubicin, vincristine, prednisolone, ifosfamide, methotrexate, VP-16, dexamethasone).

h LNH (cyclophosphamide, vindesine, bleomycin, prednisone, methotrexate and either adriamycin or mitoxantrone).

i VNCOP-B (cyclophosphamide, mitoxantrone, vincristine, etoposide, bleomycin, prednisone).

shows information about the trials, namely country of origin, age range of the patients, the chemotherapy treatment administered and the number of patients in each treatment arm.

Table 2

**Table 2:** The relative risk (or ratio) in relation to specified outcomes, comparing the rate in the G-CSF group with the rate in the non-G-CSF group

	**Relative risk and 95% confidence interval**					
	**Severe neutropenia^a^**	**Severe or clinically important infection^b^**	**Hospitalisation^c^**	**Treatment delays**		
**Trial reference**				**All delays^d^**	**Delays due to neutropenia**	**Mean hospital stay (ratio)^e^**
Pettengell *et al* (1992)	0.44 (0.23–0.85)	0.63 (0.26–1.55)	0.95 (0.51–1.77)	0.48 (0.22–1.02)	0.10 (0.02–0.43)	0.44 (P=0.01)
Gerhartz *et al* (1993)		0.73 (0.44–1.21)	0.45 (0.23–0.89)	0.35 (0.17–0.69)		0.19 (P<0.001)
Aviles *et al* (1994)		0.17 (0.07–0.37)			0.11 (0.05–0.23)	0.75 (P>0.05)
Bertini *et al* (1996)	0.47 (0.23–0.98)	0.47 (0.09–2.42)				0.53 (P<0.05)
Fridik *et al* (1997)		0.68 (0.21–2.13)				
Gisselbrecht *et al* (1997)	0.70 (0.47–1.03)	0.50 (0.31–0.81)			0.24 (0.11–0.56)	
Zinzani *et al* (1997)	0.42 (0.24–0.73)	0.25 (0.08–0.75)	0.09 (0–0.99)			
All trials	0.56 (0.42–0.74)^f^	0.57 (0.42–0.77)^f^,^g^	0.40 (0.21–0.77)^c^	0.40 (0.24–0.69)	0.20 (0.10–0.41)^g^	0.53
	0.54 (0.41–0.71)^h^	0.57 (0.42–0.76)^h^,^g^	0.55 (0.26–1.18)^c^			

a Absolute neutrophil count <0.5 × 10^9^ l^−1^.

b Defined as – ANC<0.5 × 10^9^ l^−1^ and fever ⩾37.5°C (Pettengell); severity score 2–4 (Gerhartz *et al*, 1993); not specified (Aviles *et al*, 1994); ⩾grade 3 (Bertini *et al*, 1996); described as ‘documented infection’ (Fridik *et al*, 1997); ⩾grade 2 (Gisselbrecht *et al*, 1997); described as ‘clinically relevant’ (Zinzani *et al*, 1997).

c Based on admissions due to infection except Pettengell *et al* (1992) in which all admissions were included. The pooled relative risk of 0.40 excludes the trial by Pettengell *et al* (1992) and the one of 0.55 includes this trial.

d ⩾8 days (Pettengell *et al* (1992)) or ⩾10 days (Gerhartz *et al* 1993).

e The ratio of the mean number of days in hospital in the G-CSF group to the mean in the non-G-CSF group. The *P*-values were those reported in the publication based on comparing G-CSF with the non-G-CSF group. The pooled ratio is a weighted average of the individual ratios, weighted by the total number of patients in each trial.

f Excluding the nonrandomised trial by Bertini *et al* (1996).

g Excluding the trial by Aviles *et al* (1994) because the results were based on number of cycles, not patients.

h Including the nonrandomised trial by Bertini *et al* (1996).

shows the relative risk associated with neutropenia, clinically relevant infection (defined in various ways, see footnote to table), hospitalisation and treatment delays and the ratio of the mean hospital stay in the G-CSF (or GM-CSF) group compared to those not given growth factors. There was no evidence of heterogeneity in relation to any of the outcomes (*P*>0.16).

G-CSF was associated with a statistically significant 44% reduction in the incidence of severe neutropenia (relative risk 0.56, *P*<0.001) and a 43% reduction in the number of patients with a clinically relevant infection (relative risk 0.57, *P*<0.001). As a consequence, there was a 60% reduction in the number of hospital admissions due to infection (relative risk 0.40, *P*=0.006) and if a patient on G-CSF were admitted they spent about half the time in hospital (ratio of the mean hospital stay 0.53). Administering G-CSF also had an effect on patients experiencing a treatment delay; there was a 60% reduction in the number of patients experiencing a treatment delay for any reason (relative risk 0.40, *P*<0.001) and an 80% reduction in the number of patients whose delay was due to neutropenia (relative risk 0.20, *P*<0.001).

Table 3

**Table 3:** The relative risk of having a complete/partial tumour remission or surviving to 2 or more years in the G-CSF group compared to the non-G-CSF group

	**Relative risk and 95% confidence interval**		
**Trial reference**	**Complete remission**	**Complete or partial remission**	**Survival**
Pettengell *et al* (1992)		0.98 (0.62–1.55)	1.07 (0.62–1.85), 2 years
Gerhartz *et al* (1993)	1.23 (0.79–1.93)	1.03 (0.74–1.43)	
Aviles *et al* (1994)	1.47 (0.69–3.10)		
Bertini *et al* (1996)	0.91 (0.55–1.50)		
Fridik *et al* (1997)	1.14 (0.67–1.97)		1.16 (0.66–2.04), 5 years
Gisselbrecht *et al* (1997)	0.92 (0.64–1.34)	0.99 (0.71–1.38)	1.15 (0.77–1.72), 5 years
Zinzani *et al* (1997)	1.02 (0.67–1.56)		1.02 (0.68–1.53), 2.5 years
All trials	1.07 (0.87–1.32)^a^	1.00 (0.81–1.24)	1.15 (0.83–1.60), 5 years
	1.05 (0.87–1.27)^b^		1.04 (0.75–1.44), 2 or 2.5 years

a Excluding the nonrandomised trial by Bertini *et al* (1996).

b Including the nonrandomised trial by Bertini *et al* (1996).

shows the results in relation to tumour remission and survival. There was no heterogeneity between the trials reporting on complete remission (*P*=0.90), those reporting on complete or partial remission combined (*P*=0.99) or those reporting on survival (*P*>0.90). There was no evidence that the use of G-CSF influenced tumour remission or survival; the pooled relative risks were close to unity and none were statistically significant.

### Cost-effectiveness

From the section above, in patients given G-CSF compared to those who were not, the relative risk for hospital admission due to infection was 0.4 and the ratio for the length of stay was 0.53. These estimates are used in the following cost-effectiveness analysis.

Table 4

**Table 4:** The percentage reduction in the list price of G-CSF per patient^a^ required such that the cost to the health service is less than the cost of not using it. The estimates are based on a standard dose of 5 *μ*g kg day^−1^; the estimates in brackets are based on a dose of 2 *μ*g kg day^−1^

	**Number of hospital admissions per patient**				
**Percentage of patients hospitalised (or the chance of being hospitalised) %**	**1**	**2**	**3**	**4**	**5**
5	97 (94)	95 (88)	93 (82)	90 (75)	88 (69)
10	95 (88)	90 (75)	85 (63)	80 (51)	75 (38)
15	93 (82)	85 (63)	79 (45)	70 (26)	63 (8)
20	90 (75)	80 (51)	70 (26)	61 (2)	51 (0)
25	88 (69)	75 (38)	63 (8)	51 (0)	38 (0)
30	85 (63)	70 (26)	58 (0)	41 (0)	26 (0)
35	83 (57)	66 (14)	48 (0)	31 (0)	14 (0)
40	80 (51)	61 (2)	41 (0)	21 (0)	2 (0)
45	79 (45)	56 (0)	34 (0)	11 (0)	0 (0)
50	75 (38)	51 (0)	26 (0)	2 (0)	0 (0)

a The cost of G-CSF is taken to be £4406, assuming £75 (British National Formulation, 2002) per day over 10 days in each of the five treatment cycles, and increased by 17.5%, Value Added Tax.

shows the reduction in the list price of G-CSF needed such that the health service cost becomes cheaper if it were routinely used as a primary prophylaxis than if it were not used. A relatively large proportion of patients need to be admitted several times in the absence of using G-CSF before a policy of offering it routinely becomes cost-effective. The percentage of patients in the control group that required hospitalisation due to infection was 7% in one trial (Zinzani *et al*, 1997) and 31% in another (Gerhartz *et al*, 1993). In the UK, it is about 15% after first-line therapy and 30% after second- or third-line therapy. With these estimates, the published list price of G-CSF would have to be reduced significantly for it to be worthwhile. For example, if 15% of patients were each hospitalised twice during their course of treatment, G-CSF would have to be purchased at a cost that is 85% lower than the list price for it to be cost-effective, that is £660 compared to £4406. The required reduction in the list price is less if the dose of G-CSF can be reduced to 2 *μ*g kg day^−1^ (63%). Similarly, the higher the chance of an individual patient being hospitalised the less the reduction in the list price of G-CSF.

## DISCUSSION

The results of the meta-analyses show that the use of HGFs such as G-CSF has a significant effect on several important clinical outcomes associated with the management of patients with aggressive NHL. They result in far fewer patients with neutropenia and as a consequence fewer patients with infection, fewer who are hospitalised due to infection and fewer whose chemotherapy treatment has to be delayed. There was however no evidence of an improvement on tumour remission or survival.

Our simple cost-effectiveness analysis, based on direct costs alone, suggests that using G-CSF as a primary prophylaxis for chemotherapy-induced neutropenia would be more expensive to the service provider than not using it, a similar conclusion found by others in Canada and Italy (Zagonel *et al*, 1994, Dranitsaris *et al*, 1997). However, the analysis will to some extent underestimate the cost-effectiveness of using G-CSF because we only included hospital admissions due to febrile neutropenic events but there will be patients with infection who require treatment that are not hospitalised and we did not include the cost of having a treatment delay due to neutropenia. When indirect costs have been included in other cost-effectiveness analyses, for example loss of earnings in patients unable to work because of a neutropenic event, it has been concluded that HGFs can be cost-effective (Dranitsaris *et al*, 1997). The inclusion or exclusion of such indirect costs in patients with haematological malignancies needs further consideration together with the quality of life benefits, as recommended by the 1998 Office of Health Economics report on NHL.

The evidence shows that the routine use of HGFs is worthwhile. Although they may seem to be expensive to the health service, it would be unsatisfactory to choose financial saving over patient health.

In 1999, there were 9014 new cases of NHL in the UK (ONS, 2001) and it is estimated that the incidence is rising by about 4% per annum (Office of Health Economics, 1998). As a consequence, the number of patients with high-grade NHL is likely to increase. Currently, HGFs are not administered routinely to such patients, although it is possible that patients with mild neutropenic fever could be managed at home with oral antibiotics. Using HGFs is clearly clinically worthwhile but the costs need to be significantly reduced for there to be direct financial savings to the health service.

## References

[bib1] Aviles A, Diaz-Maqueo JC, Talavera A, Nambo MJ, Garcia EL (1994) Effect of granulocyte colony-stimulating factor in patients with diffuse large cell lymphoma treated with intensive chemotherapy. Leuk Lymphoma 15: 153–157 7532056 10.3109/10428199409051691

[bib2] Bertini M, Freilone R, Vitolo U, Botto B, Ciotti R, Cinieri S, Di Nova A, Di Vito F, Levis A, Orsucci L, Pini M, Rota-Scalabrini D, Todeschini G, Resegotti L (1996) The treatment of elderly patients with aggressive non-Hodgkins lymphoma: feasibility and efficacy of an intensive multi-drug regimen. Leuk Lymphoma 22: 483–493 8882962 10.3109/10428199609054787

[bib3] British National Formulation (2002) (number 43). London: British Medical Association & the Royal Pharmaceutical Society of Great Britian

[bib4] Dranitsaris G, Altmayer C, Quirt I (1997) Cost–benefit analysis of prophylatic granulocyte colony-stimulating factor during CHOP antineoplastic therapy for non-Hodgkin's lymphoma. Pharmacoeconomics 11(6): 566–577 10173030 10.2165/00019053-199711060-00005

[bib5] Fridik MA, Greil R, Hausmaninger H, Krieger O, Oppitz P, Stoger M, Klocker J, Neubauer M, Helm W, Pont J, Fazeny B, Hudec M, Simonitsch I, Radaszkiewicz T (1997) Randomised open label phase III trial of CEOP/IMVP-Dexa alternating chemotherapy and filgrastim *versus* CEOP/IMVP-Dexa alternating chemotherapy for aggressive non-Hodgkins lymphoma (NHL). A multi-center trial by the Austrian Working Group for Medical Tumour Therapy. Ann Hematol 75: 135–140 9402845 10.1007/s002770050330

[bib6] Gerhartz HH, Engelhard M, Meusers P, Brittinger G, Wilmanns W, Schlimok G, Mueller P, Huhn D, Musch R, Siegert W, Gerhartz D, Hartlapp JH, Thiel E, Huber C, Peschl C, Spann W, Emmerich B, Schadek C, Westerhausen M, Pees H-W, Radtke H, Engert A, Terhardt E, Schick H, Binder T, Fuchs R, Hasford J, Brandmaier R, Stern AC, Jones TC, Ehrlich HJ, Stein H, Parwaresch M, Tiemann M, Lennert K (1993) Randomized, double-blind, placebo-controlled, phase III study of recombinant human granulocyte-macrophage colony-stimulating factor as adjunct to induction treatment of high-grade malignant non-Hodgkin's lymphoma. Blood 82: 2329–2339 7691256

[bib7] Gisselbrecht C, Haioun C, Lepage E, Bastion Y, Tilly H, Bosly A, Dupriex B, Marit G, Herbrecht R, Deconnick E, Marolleau JP, Yver A, Dabouz-Harrouche F, Coiffier B, Reyes F (1997) Placebo-controlled phase III study of lenograstim (glycosylated recombinant human granulocyte colony-stimulating factor in aggressive non-Hodgkins lymphoma: factors influencing chemotherapy administration. Leuk Lymphoma 25: 289–300 9168439 10.3109/10428199709114168

[bib8] Office for National Statistics (ONS) (2001) Registrations, England 1999. Series MB1, no. 30. London: OBS

[bib9] Office of Health Economics (1998) The Economic Aspects of Non-Hodgkin's Lymphoma. London: OHE

[bib10] Pettengell R, Gurney H, Radford JA, Deakin DP, James R, Wilkinson PM, Kane K, Bentley J, Crowther D (1992) Granulocyte colony-stimulating factor to prevent dose-limiting neutropenia in non-Hodkin's lymphoma. A randomised controlled trial. Blood 6: 1430–1436 1381626

[bib11] Toner GC, Shapiro JD, Laidlaw CR, Rischin D, Millward MJ, Wolf M, Januszewicz H, Mitchell SV, Curran AC, Matthews JP, Bishop JF (1998) Low-dose *versus* standard-dose lenograstim prophylaxis after chemotherapy: a randomized, crossover comparison. J Clin Oncol 16(12): 3874–3879 9850033 10.1200/JCO.1998.16.12.3874

[bib12] Whitehead A, Whitehead J (1991) A general parametric approach to the meta-analysis of randomised clinical trials. Stat Med 10: 1665–1677 1792461 10.1002/sim.4780101105

[bib13] Zagonel V, Babare R, Merola MC, Talamini R, Lazzarini R, Tirelli U, Carbone A, Monfardini S (1994) Cost–benefit of granulocyte colony-stimulating factor administration in older patients with non-Hodgkin's lymphoma treated with combination chemotherapy. Ann Oncol 5(Suppl 2): S127–S132 10.1093/annonc/5.suppl_2.s127 7515645

[bib14] Zinzani PL, Pavone E, Storti S, Moretti L, Fattori PP, Guardigni L, Falini B, Gobbi M, Gentilini P, Lauta VM, Bendandi M, Gherlinzoni F, Magagnoli M, Venturi S, Aitini E, Tabanelli M, Leone G, Liso V, Tura S (1997) Randomized trial with or without granulocyte colony-stimulating factor as adjunct to induction VNCOP-B treatment of elderly high-grade non-Hodgkin's lymphoma. Blood 89: 3974–3979 9166835

